# Physical and psychological effects of different temperature-controlled breast prostheses on patients with breast cancer during rehabilitation: a randomized controlled study (CONSORT)

**DOI:** 10.1097/MD.0000000000019616

**Published:** 2020-03-27

**Authors:** Jiajia Qiu, Lichen Tang, Lijin Huang, Shengqun Hou, Jie Zhou

**Affiliations:** a Department of Nursing Administration, Shanghai Cancer Center; b Department of Oncology, Shanghai Medical College; c Department of Breast Surgery, Shanghai Cancer Center, Fudan University, Shanghai, China.

**Keywords:** breast cancer, breast prostheses, physical, psychological, randomized controlled trial

## Abstract

**Background:**

Breast loss causes negative influence on women physically, psychologically, and socially. Breast prosthesis can improve patient's figure externally, increase self-confidence, thus improving quality of life (QOL). Prospective study of different breast prostheses has not yet been performed in China. Our objective was to evaluate the QOL of patients wearing different types of breast prostheses and to compare the physical and psychological effects of different temperature-controlled breast prostheses on patients.

**Methods:**

Thirty patients with breast cancer were recruited through the Yankang E-follow-up Platform at the Department of Breast Surgery of Fudan University, Shanghai Cancer Center and were randomized into either intervention or control group. Random number tables were used in this study for randomization. In the first 6 weeks of the study, self-adhesive breast prostheses and conventional breast prostheses had been used in the intervention and control group, respectively. In the later 6 weeks, the breast prostheses used were switched into another kind. Several dimensional parameters including skin conditions, breast prosthesis knowledge, breast prosthesis knowledge, QOL, and body image were examined by different questionnaires in the end of both 6th and 12th week.

**Results:**

There were no significant difference in QOL and body image between the 2 groups during 6th and 12th week. At the 6th week of the study, patients of the intervention group preferred to the self-adhesive breast prosthesis, indicating that the self-adhesive breast prosthesis seemed more likely to feel like part of their body, while prosthesis cleaning remaining their biggest concern. At the end of 12th week, in comparison with the number at 6th week, more patients in both groups were willing to choose self-adhesive breast prosthesis.

**Conclusions:**

We conclude that women are satisfied with the temperature-controlled breast prosthesis and are more willing to choose self-adhesive breast prostheses although cleaning remains a problem. In China, patients still lack information about breast prostheses. Therefore, specialist breast nurses should provide comprehensive information about breast prostheses, assist patients in selecting suitable breast prostheses, collect feedback about the prostheses, and reduce each patient's physical and mental discomfort.

## 1 Introduction

Breast cancer is the most common type of malignant tumor among women. From 2003 to 2007, the total incidence of female breast cancer at the 32 cancer registries in China was 41.64 cases per 100,000 people, ranking as the most common female cancer with highest incidence rate in Shanghai (68.58/100,000 people).^[1]^ The incidence of female breast cancer in both urban and rural areas of China is increasing every year.^[2]^ In 2007, the overall incidence of breast cancer in Shanghai was 2.25-fold higher than in 1988.^[3]^ Breast cancer has become the first-rank killer of women in China.^[4]^

The comprehensive treatment of early breast cancer mainly involves surgery combined with chemotherapy, endocrinology, radiotherapy, and biological targeted therapy. Surgery is a first-line treatment for early-stage breast cancer.^[5]^ Breast-conserving surgery has become the first priority for early-stage breast cancer in Europe and the United States. However, in China, limited knowledge about breast preservation has led to less affected population receiving breast-conserving surgery,^[6]^ therefore, mastectomy is still the primary choice for breast cancer treatment.^[7]^ A total mastectomy brings problems to breast cancer patients who lose their breast after surgery, including psychological issues such as fear, depression, anxiety, and pessimistic despair.^[8,9]^ Although, comprehensive treatments may improve long-term survival rates of patients, but the psychological stress on women caused by mastectomy is far-reaching.^[10]^ Psychological reactions may vary according to age, educational level, economic status, and residence location.^[11]^ Patients undergoing mastectomy may experience challenges related to their physical disability, illness, family life, and society during their rehabilitation period. These challenges can considerably and negatively impact patients’ self-image and psychophysiological health and may seriously affect quality of life (QOL). Modified radical surgery can result in decreased social interactions^[12]^ due to postoperative chest shape changes and thoracic scoliosis caused by the imbalance of the chest. The absence of the breast has a significant negative impact on feminine feelings and self-esteem,^[13]^ leading to self-abasement in their sexuality and self-image.^[14]^ The link between self-esteem and duration of untreated depression might have a substantial impact on the clinical outcomes of depressed individuals.^[15]^ Most patients who undergo a modified radical mastectomy have a sense of unease and feel physically imbalanced due to the absence of the breast, which leads to a decline in their QOL and emotions.^[16]^

For breast cancer patients who are unable or unwilling to undergo breast reconstruction surgery, a breast prosthesis becomes a good option.^[17]^ Wearing a breast prosthesis can not only make up for the physical disability to improve the external body image but also can protect the wound site from external strikes and prevent chest pain. Meanwhile, it can also prevent scoliosis of the spine caused by long-term imbalance of the body. More importantly, patients can increase their self-confidence and enjoy their family lives and social activities.^[18]^ A good breast prosthesis can benefit a woman with an absent breast in regaining her body image, femininity and psychological well-being.^[19]^ At present, 2 types of breast prostheses are commonly used. The conventional breast prosthesis that is placed inside a bra and does not directly adhere to the skin may affect some daily activities, such as doing sports and housework. The adhesive breast prosthesis adheres to the skin and can be worn all night without a bra; however, it may cause a certain degree of skin irritation.^[20]^ Thijs-Boer et al^[20]^ investigated patients undergoing radical mastectomy in the Netherlands and found that patients using breast prostheses were concerned about the prosthetic's fit, convenience, local skin irritation, and the ability to disguise their surgical scar. A study by Kubon et al^[21]^ reported that adhesive breast prostheses have more advantages than conventional ones in terms of comfort, aesthetics, and psychological perceptions.

Experimental research into different breast prostheses has not yet been performed in China. As professional medical providers, however, we have little evidenced information for our patients. We want to know whether different breast prosthesis could predict QOL, and whether the feelings of patients with different breast prosthesis change over time. By evaluating the QOL of patients wearing different types of breast prostheses as well as comparing the physical and psychological effects of different temperature-controlled breast prostheses, this study was to collect evidence to serve patients who undertake mastectomy. The research objectives were to find out,

(1) whether patients were satisfied with different temperature-controlled breast prostheses physically and psychologically.
(2) QOL of patients wearing different types of breast prostheses.
(3) and knowledge regarding breast prostheses in breast cancer patients who loss their breast

## 2 Materials and methods

### 2.1 Ethical approval

This study was approved by the Scientific and Ethical Committee of the Shanghai Cancer Center, Fudan University. All methods were performed in accordance with the relevant guidelines and regulations. Written informed consent was obtained from all participants before data collection. The individuals discussed in this manuscript have given written informed consent to publish these details.

### 2.2 Study population

This study was approved by the Scientific and Ethical Committee of the Shanghai Cancer Center, Fudan University. Written informed consent was obtained from all participants before data collection. From November 2016 to February 2017, 30 patients with breast cancer were recruited through the Yankang E-follow-up Platform at the Department of Breast Surgery of Fudan University, Shanghai Cancer Center. The inclusion criteria included patients

(1) undergoing unilateral mastectomy due to breast cancer confirmed by histological examination;
(2) had undergone mastectomy at least 6 months before the start of the study or had completed radiation therapy at least 2 months before;
(3) without evidence of postoperative relapse;
(4) wearing conventional (non-adhesive) breast prostheses;
(5) without abnormal skin or skin lesions;
(6) without progressive lymphedema; and
(7) interested in conventional and self-adhesive breast prostheses.

Exclusion criteria were as follows: patients

(1) with incomplete healing of their surgical wounds;
(2) undergoing chemoradiotherapy or had received chemoradiotherapy less than 2 months before the beginning of the study;
(3) with skin conditions that do not meet the requirements;
(4) whose remaining breast is not within the study's size range;
(5) with significant life changes during the study, including divorce, unemployment or depression;
(6) relapsed during the observation period; and
(7) had a reaction to the first skin test and unable to receive the second skin test.

### 2.3 Study methods

This study was a randomized controlled experimental study. The research subjects who met the inclusion criteria were randomized into an intervention group or control group. Two types of temperature-controlled breast prostheses were applied to patients in each group. A 12-week crossover randomized controlled study was conducted. In the first 6 weeks, the intervention group used self-adhesive breast prostheses, while the control group used conventional breast prostheses. The following parameters were examined: skin condition, perception of breast prosthesis, feelings when wearing breast prosthesis, QOL, and body image. In the latter 6 weeks, the treatment each group used was switched. In the 12th week, the above parameters were examined again.

#### 2.3.1 Randomization methods

Random number tables were used in this study for randomization. The research subjects were numbered, and then a random number for each research object was sequentially obtained in the same direction starting from any number in the random number table. The random number was divided by the number of the groups to find the remainder. If it was divisible, then the number of the groups was considered as the remainder. Otherwise, the remainder was used for grouping.

### 2.4 Research tools

#### 2.4.1 Demographic survey form

This included general and medical information for each patient, including age, occupation, education level, marital status, religion, economic status, household income, payment methods for medical expenses, operation times, surgical methods, and treatments received.

#### 2.4.2 Objective parameter measurement

Scars and skin conditions: these examinations were performed by the same investigator.

#### 2.4.3 Survey of knowledge about breast prosthesis

A self-designed questionnaire covering a total of 11 items was used, including sources the patients used to obtain information on breast prostheses, reasons for choosing the breast prosthesis, its type and price, and the feelings about wearing the breast prosthesis.

#### 2.4.4 Survey of comfort and practicality of breast prostheses

A self-designed questionnaire covering a total of 10 items regarding breast prostheses was used, including skin adhesion, practicality in daily life, maintainability, comfort, natural fit, contact, safety, and effects on the shoulder and back.

#### 2.4.5 Quality of life instruments for cancer patients: breast cancer (QLICP-BR)

This scale^[22]^ was designed by Zhang Dongmei and Wan Chonghua. Considering Chinese culture, the QLICP-BR selected the following 37 items: 6 items in physical functional dimensions (PH), 12 items in psychological functional dimensions (PS), 8 items in symptoms and side effects dimensions (ST), 10 items in social functional dimensions (SOs) and 1 item in overall health condition. Items 5 to 10 and 27 to 32 are positive items and the rest are reverse items. Each item was scored as follows: no (1), slight (2), some (3), fair (4), and considerable (5). The reliability of the QLICP-BR scale has been confirmed among Chinese patients with tumors in terms of validity, reliability, and responsiveness.

#### 2.4.6 Body image scale (Chinese version)

The body image scale is a self-assessment scale designed to assess cancer patients’ perceptions of their appearance and identify any changes to those perceptions resulting from a disease or a treatment. The scale was developed by Hopwood, P. in 2001^[23]^ and was translated into Chinese by Prof Fang Suyi.^[24]^ The scale includes 10 items and the Cronbach Alpha value was 0.90. The scoring method is as follows: “not at all”: 0 points, “slight”: 1 point, “fair”: 2 points, and “considerable”: 3 points. A higher score is associated with a worse body image.

### 2.5 Statistical analysis

SPSS 18.0 was used for the statistical analysis in this study. Means, standard deviations, and percentages were used to describe the basic conditions of the breast cancer patients, their body image and QOL scores, knowledge regarding breast prostheses, and perceived comfort and practicality of the breast prostheses. The chi-square test was used to compare the comfort and practicality of the breast prostheses, and the *t*-test was used to compare the scores for QOL and body image between the 2 groups.

## 3 Results

### 3.1 Participant flow

**Figure 1:**
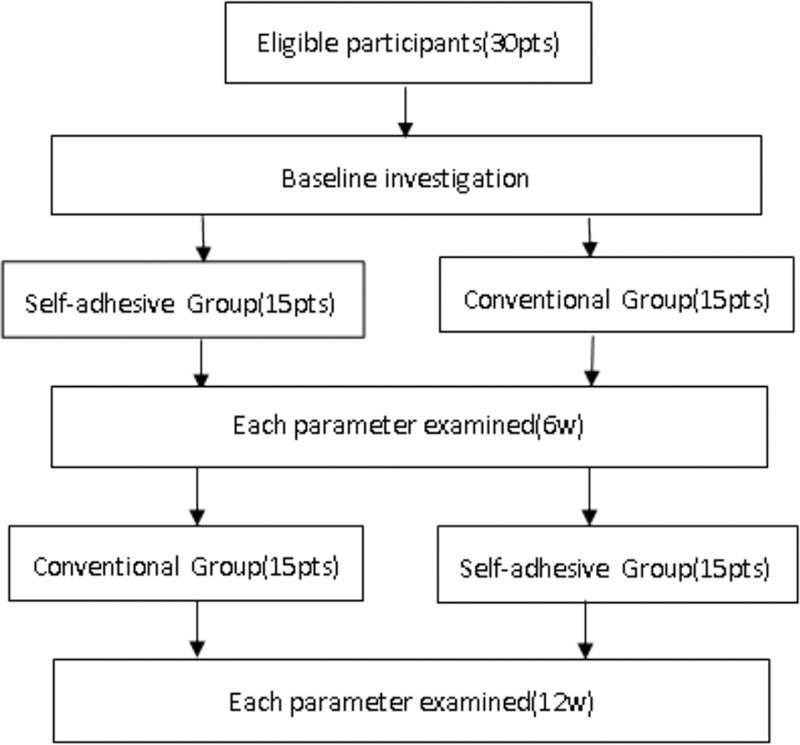
Participants flow.

Thirty participants were recruited and randomized into an intervention group or control group. In the first 6 weeks, 15 participants in the intervention group used self-adhesive breast prostheses, while 15 participants in the control group used conventional breast prostheses. Related parameters were examined. In the latter 6 weeks, the treatment each group used was switched. 15 participants in each groups were examined by the same parameters again in the 12th week (Fig. 1).

### 3.2 Baseline data of the 2 groups of patients

#### 3.2.1 Baseline characteristics of the 2 groups of patients

The average age of the patients was 48.5 years old, and the average duration of disease was 34.9 months. In all, 66.7% of the patients had a bachelor's degree or above, 66.7% of the patients had a monthly income between RMB 5000 and 15,000 Yuan, 90% of the patients had medical insurance, 70% of the patients received chemotherapy, and 26.7% of the patients received radiotherapy. There was no significant differences in baseline information, medical information, and initial skin conditions between the 2 groups.

#### 3.2.2 Knowledge about breast prostheses in the 2 groups of patients

The initial point at which the patients began to wear breast prostheses was 4.37 months after surgery. Based on the baseline survey, 73.3% of the patients were willing to wear “bra-type” breast prostheses, and 66.7% of the patients preferred breast prostheses that cost less than 1000 Yuan. Additionally, 56.7% of the patients wore their breast prostheses all day until bed time and 100% of these patients believed that the breast prostheses enhanced their self-image. While 53.3% of them thought that their breast prostheses improved their sexuality, only 6.7% of them had thorough knowledge about their breast prostheses. In addition, 86.7% of the patients believed that it was necessary to obtain information about breast prostheses through their medical providers.

There was no significant difference in knowledge about breast prostheses between the 2 groups

#### 3.2.3 Perceptions of comfort and practicality of breast prostheses in the 2 groups

For conventional bra-type breast prostheses, 96.7% of the patients believed that it was easy to wear breast prosthesis; 80% of the patients thought it did not require a lot of time to wear; 70% thought that it was easy to clean breast prosthesis; 13.3% of patients thought that wearing this type of breast prosthesis could cause localized itching, discomfort in the shoulder and back, and an embarrassing sound; 60% of patients thought that the weight of the breast prosthesis was similar to their own breast; 70% of them said that the breast prosthesis was enough to cover the surgical scar; 63.4% of patients felt that the breast prosthesis seemed to integrate with their body; and 50% of the patients were satisfied with their conventional breast prosthesis. Comparisons of baseline data between the 2 groups were not significantly different.

#### 3.2.4 QOL and body image in the 2 groups of patients

Baseline surveys in both groups of patients revealed high scores in all dimensions of QOL, suggesting that the QOL among patients was better (a higher score is associated with a better QOL with this scale). The body image score exceeded the average score, suggesting that body image among the patients was not very satisfying (a higher core is associated with a worse body image). There was no significant difference between the 2 groups of patients.

### 3.3 Comparison between the 2 groups of patients in the 6th week

#### 3.3.1 Comparison of skin conditions between the 2 groups of patients

For the first 6 weeks, the intervention group used adhesive breast prostheses and the control group used conventional ones. During this 6-week period, 2 different breast prostheses did not cause any complications such as rashes, redness or ulceration of the patients’ skin in either group (Table 1).

**Table 1:**
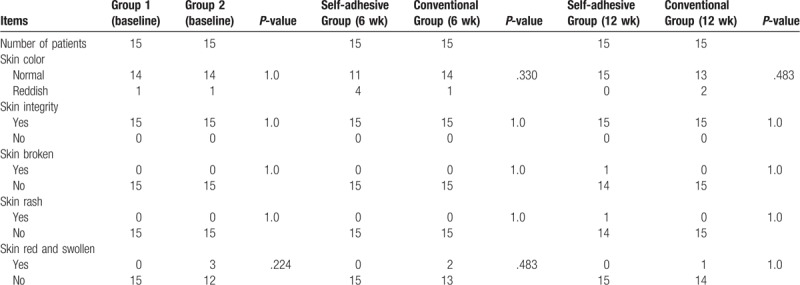
Comparison of skin condition between the 2 groups of patients.

#### 3.3.2 Comparison of knowledge about breast prosthesis in the 2 groups of patients

By the 6th week of the study, patients in the intervention group were more willing to choose the adhesive breast prosthesis (Table 2).

**Table 2:**
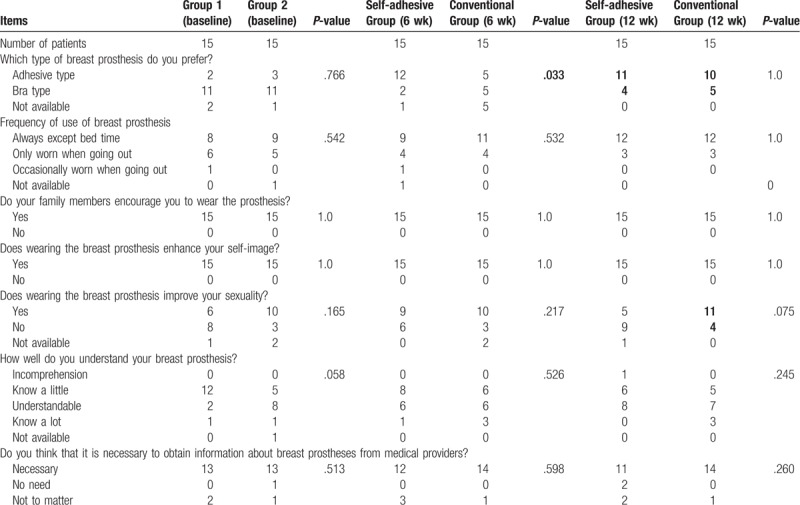
Comparison of breast prosthesis knowledge in the 2 groups of patients.

#### 3.3.3 Comparison of the comfort and practicality of breast prostheses in the 2 groups

During this 6-week period, more patients in the control group complained about shoulder and back discomfort. More patients in the intervention group indicated that the breast prosthesis seemed to be a part of their body, but they were concerned about the cleaning issues (Table 3).

**Table 3:**
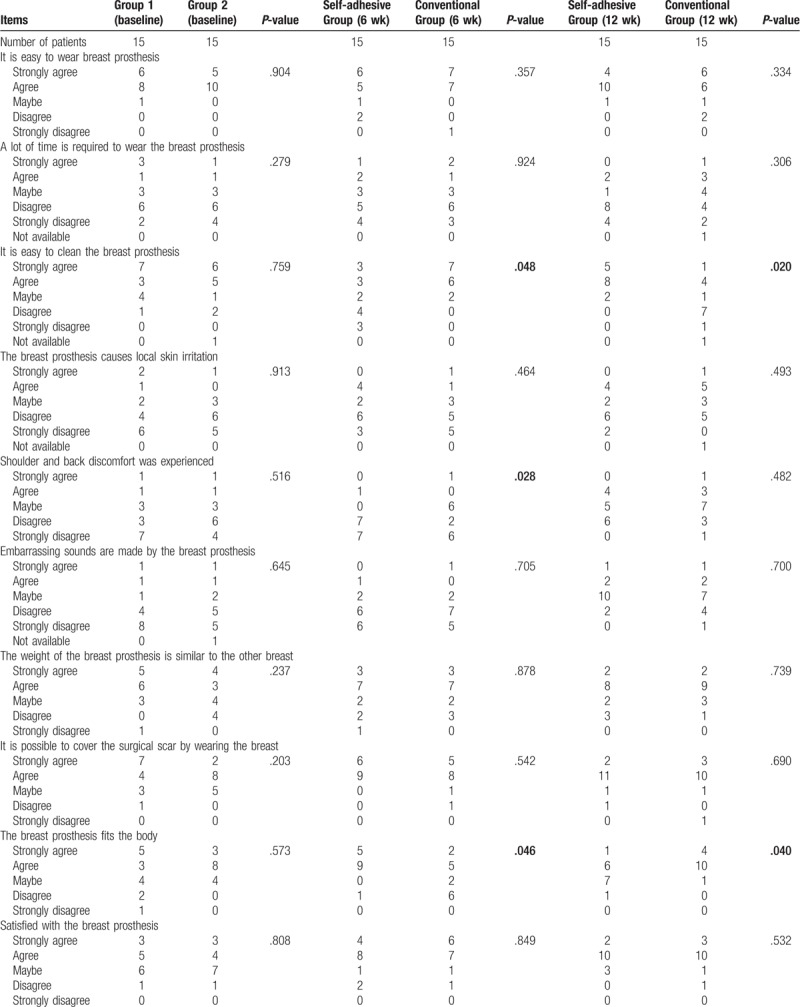
Comparison of comfort and practicality between the 2 groups of patients.

#### 3.3.4 Comparison of QOL and body image in the 2 groups

During this 6-week period, there was no significant difference in QOL and body image between the 2 groups (Table 4).

**Table 4:**
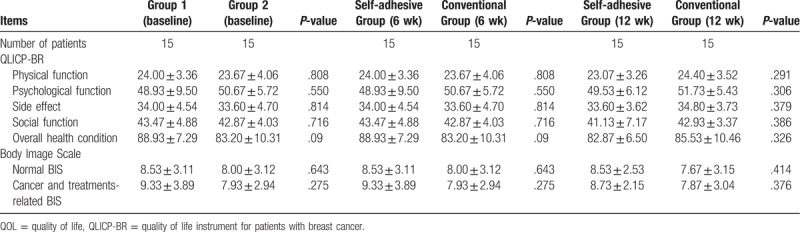
Comparison of QOL and body image between the 2 groups of patients.

### 3.4 Comparison between the 2 groups of patients in the 12th week

#### 3.4.1 Comparison of skin conditions between the 2 groups

In the latter 6 weeks of the study, the intervention group used conventional breast prostheses and the control group used adhesive ones. During this 6-week period, neither type of breast prosthesis caused complications such as rashes, redness or ulceration of the patients’ skin (Table 1).

#### 3.4.2 Comparison of knowledge about breast prosthesis in the 2 groups

In the 12th week, although there was no significant difference between the 2 groups in their choices of breast prostheses, more patients in both groups were willing to choose to wear adhesive breast prostheses. Although there was no significant difference in responses regarding whether the use of the breast prosthesis improved the sexuality, more patients in the control group who wore the adhesive type thought that the breast prosthesis could improve their sexuality. The number of patients who reported improved sexuality increased during the later 6 weeks (Table 2).

#### 3.4.3 Comparison of the comfort and practicality of the breast prostheses in the 2 groups

By the 12th week of the study, more patients in the control group reported that the breast prostheses were more closely fit to their bodies. Meanwhile, patients using self-adhesive breast prostheses complained that they were more difficult to clean (Table 3).

#### 3.4.4 Comparison of QOL and body image in the 2 groups

During the 12-week period, there was no significant difference in QOL and body image between the 2 groups (Table 4).

## 4 Discussion

The present study was the first randomized control trial with regard to different types of breast prostheses in mainland China. Currently, we have little clinical evidence to provide breast prostheses related professional information to breast cancer patients.

The QOL among patients was better, but the body image score was not very satisfying, there was no significant difference between the 2 groups of patients during the whole study. Women in our study were satisfied with the temperature-controlled breast prosthesis. In terms of physiology, a radical mastectomy can cause an asymmetrical trunk, shoulders and waist, excessive forward trunk lean, and asymmetrical scapulae.^[25,26]^ Psychologically, studies have shown that breast loss can lead to

(1) loss of attractiveness;
(2) decreased self-esteem; and
(3) loss of intimate function.^[27–28]^

They felt embarrassed and as if their bodies were incomplete. They fear to be rejected and to start new relationships. Nearly half of the subjects in the study by Duarte et al^[29]^ reported that they did not want their partners to touch them, they did not want to take off their clothes, and they needed to wear clothes and bras during intimate situations.

The overall rehabilitation protocol after total mastectomy includes the use of a breast prosthesis. It refers to the placement of pseudo-materials in clothes that can be molded into the shape of a breast.^[30]^ The natural and self-adhesive breast prostheses owns a patent for Comfort+ technology were selected in this study. Natural-style breast prostheses^[20,30]^ are composed of 2 silicones with different levels of softness; these must be placed in a bra. The adhesive type is directly adhered to the chest and thus can be used during physical activities.^[20,30]^ This type can be firmly attached to the body and used without a bra.

Non-adhesive natural-style breast prostheses must be worn with a bra and do not directly touch the skin. Their disadvantages include that they can easily change positions and that they put weight on the shoulders.^[20]^ During the first 6-week period, more patients in the control group complained about shoulder and back discomfort. Self-adhesive breast prostheses can be applied directly to the skin and do not require bras. The advantage herein is that the chest wall bears the weight rather than the shoulders. This type is easy to wear and requires less time,^[20]^ but it can sometimes cause skin rashes.^[20,31]^ A study by Thijs-Boer et al^[20]^ showed that most patients prefer adhesive breast prostheses mainly because of their fit. Moreover, a preference for adhesive breast prostheses was not associated with age. Adhesive breast prostheses, however, need to be cleaned and dried every day to prevent sweat stains. Many patients complained that this maintenance was troublesome and indicated that this might affect their decision to use them. Munstedt et al^[32]^ reported that 90.7% of patients who underwent radical surgery chose adhesive breast prostheses. Conventional silicone breast prostheses sometimes make noises when struck,^[32]^ which rarely happens to adhesive breast prostheses. Moreover, non-adhesive breast prostheses can easily shift with body movements. This can be a problem for patients.^[20,33,34]^ Our study showed that neither the adhesive nor the natural breast prostheses used in this study caused complications such as skin rashes, redness, or ulceration of the patient's skin. The subjects indicated that the natural type of breast prosthesis is easy to wear, maintain and clean, but it must be worn with a relatively high-quality bra. The natural breast prosthesis can easily slide up or down and is not as good as an adhesive breast prosthesis in appearance and feel. In contrast, the adhesive breast prosthesis has a more realistic feel, a more appealing appearance and is compatible with more types of bras. It can also be worn when exercising. Research subjects were more willing to choose adhesive breast prostheses and commented that they were more likely to feel like a part of the body. Patients feel more flexible and less restricted as adhesive breast prostheses do not require a bra and are less noisy. Some patient statements included “Wearing an adhesive breast prosthesis is very comfortable during exercises. It is perfectly integrated with my body. When I’m exercising, it allows me to jump freely with a sense of breast bouncing. I feel confident.” “My chest bears the weight of my adhesive breast prosthesis. I feel like myself after wearing it for a long time, and low-cut tops enhance my self-confidence.” However, some patients said that “An adhesive breast prosthesis is more troublesome to wear. It always takes time to adjust its position relative to the real one in front of a mirror. Sometimes it hurts when I take them off and it is inconvenient to clean.”

Comfort is the most important factor affecting prosthetic breast use and satisfaction.^[27,35–38]^ The weight and texture of breast prostheses is an important factor affecting comfort. Heavy prostheses can cause shoulder pain. The most commonly used material is silicone, which is hot in summer and cold in winter. It causes sweating in summer and sometimes makes noises.^[20,27,31,39]^ The 2 types of breast prostheses used in this study are made with comfort temperature control technology that allows them to absorb heat from the body to keep the chest cool and comfortable during hot summer days. The technology also allows the prostheses to absorb heat from the body and return it to the body during cold winter days to keep the chest warm. Adjustable temperature technology ensures comfort when wearing this type of prosthesis. Moreover, the adhesive type of breast prosthesis is lightweight, only about 30% the weight of a conventional one. In our study, 73.3% of patients were willing to wear a “bra-type” breast prosthesis during the baseline survey, and half of them were satisfied with this type of prosthesis. Interestingly, in the 6th and 12th weeks, 83.3% of the patients were satisfied with the breast prosthesis they used, regardless of whether it was the adhesive or natural prosthesis. This is consistent with the findings of Thijs-Boer et al. Their study showed that the first time a conventional type of breast prosthesis was used, satisfaction was higher, but after using an adhesive breast prosthesis, satisfaction with the traditional type of breast prosthesis decreased because the adhesive type made people feel more like a part of their body. Women who had previously worn conventional breast prostheses reported that they were more satisfied with customized breast prostheses because they were more comfortable to wear and fit better. Thus, their feelings of being disabled were relieved.

The use of a breast prosthesis can improve a patient's psychosocial health.^[40]^ Chinese researchers have analyzed factors affecting the use of breast prostheses. These factors include

(1) reconstructive surgery;
(2) comfort;
(3) limitations on appearance and circumstances;
(4) price;
(5) psychological factors; and
(6) supportive information.^[41]^

The physical properties and prices of breast prostheses are important, but the availability of professional information about breast prostheses in China is extremely lacking. A lack of information is associated with a reduced frequency of breast prosthesis use^[19,27,35,38,42,43]^ and patient dissatisfaction. Sufficient information and support regarding breast prostheses affects the satisfaction of breast prosthesis users. Only 6.7% of patients in our study's baseline survey had sufficient knowledge regarding breast prostheses; 86.7% of patients believe that it is necessary to obtain information about breast prostheses through their medical providers.

Currently, information about breast prostheses is provided by manufacturers.^[44]^ Although manufacturers may provide trainings for breast prosthesis technicians, the technicians may not have received systematic training and expertise in the field of cancer and patient care. Thus, they may not have the professional skills to deal with female patients facing psychological and emotional problems in their battle with cancer.^[45]^ Nurses who specialize in breast care are the best assistants. They play an important role in breast prosthetic education. They can not only provide patients with professional knowledge and support about breast prostheses but can also coordinate with patients and breast prosthetic technicians. Provided at the right time in the right environment with the right method, professional information could then help patients to better prepare physically and mentally and make cost-efficient decisions.

Nurses can help patients adjust to physical changes and help them to respond positively through encouragement and education. Specialist breast nurses should be familiar with information about and resources related to breast prostheses so that they can provide education and support for patients as needed. Although a breast prosthesis can never completely replace an absent breast, proper use of a breast prosthesis can help patients adapt to changes due to a cancer diagnosis and any resulting changes in body image. Meanwhile, the use of a prosthesis can prevent long-term complications, including dropped shoulders syndrome, and ultimately improve the QOL of patients. Specialist breast nurses can professionally evaluate breast prostheses every 2 years to adapt them to changes in body posture or to the remaining breast's tissue caused by treatment or age.^[40]^

## 5 Study limitations

The present study has some limitations. The sample size was really small and this was the major shortcoming of this study. Limited by sample size (voluntary patients who undertook mastectomy), geographical locations (in 1 cancer center) and climate (in winter), the study cannot fully reflect the whole picture of mainland China. A single service is usually insufficient to satisfy a complicated demand, just like a Web Service Composition paradigm is introduced as a core task of integrating multiple services to generate a value-added composite web service.^[46]^ We plan to enlarge the sample size and carry out studies in different geographical locations and seasons for future research, so as to provide adequate information and support to breast cancer patients who lose breast and in need of breast prostheses.

## 6 Conclusions

As a replacement for a real breast, a breast prosthesis can increase a woman's self-esteem and self-confidence, restore her social credibility, sense of belonging, and better participation in sports. Women are satisfied with the temperature-controlled breast prosthesis and are more willing to choose adhesive breast prostheses because they are more likely to feel like a part of the body. However, they also need careful maintenance. In China, patients still lack information about breast prostheses. Therefore, specialist breast nurses should provide such information, assist patients in selecting suitable breast prostheses, collect feedback about the prostheses, and reduce patient's physical and mental discomfort.

## Acknowledgments

The authors would like to thank the women who participated in this research and shared their experiences.

## Author contributions

**Conceptualization:** Jiajia Qiu, Lichen Tang, Shengqun Hou.

**Formal analysis:** Jiajia Qiu.

**Investigation:** Lijin Huang, Jie Zhou.

**Methodology:** Jiajia Qiu, Lijin Huang.

**Supervision:** Shengqun Hou.

**Writing – original draft:** Jiajia Qiu, Lichen Tang.

**Writing – review & editing:** Jiajia Qiu, Lichen Tang.

## References

[1] Huang ZZ Chen WQ Wu CX Incidence and mortality of female breast cancer in China–a report from 32 Chinese cancer registries, 2003–2007. Tumor 2012;32:435–9.

[2] Li N Zheng RS Zhang SW Analysis and prediction of breast cancer incidence trend in China. Chin J Prevent Med 2012;8:703–7. 23157863

[3] Huang ZZ Chen WQ Wu CX The trends of female breast cancer incidence and mortality in Beijing, Shanghai, Linzhou and Qidong in China. Tumor 2012;32:605–8.

[4] Li MF Wang XH Zhao XY Research of breast cancer incidence. J China Tradit Chin Med Inf 2011;15:76.

[5] Chen W Wang CS Zhang W Comparison of breast-conserving therapy and mastectomy. Chin Prim Med 2013;2:255–7.

[6] Zhang BN Yu ZH Key problems in breast-conserving surgeries. Chin J Oncol 2001;23:523–4.

[7] Zhang BN Zhang B Tang ZH 10-year changes and development of surgical treatment for breast cancer in China. Chin J Oncol 2012;8:582–7. 10.3760/cma.j.issn.0253-3766.2012.08.005 23158990

[8] Zhang YS Liu YJ A review for the surgical management of breast cancer and the latest developments. Mod Oncol 2015;5:719–22.

[9] Song YL Zhang KM Development of psychological nursing in postoperative breast cancer patients who lost their breasts. Med Equip 2015;2:125–6.

[10] Sun LQ Cognitive study on prostheses wearing in postoperative breast cancer patients who lost their breasts. J Qilu Nurs 2010;16:48–9.

[11] Xie SH Physical and psychological influence of breast loss on postoperative breast cancer patients and nursing strategy. Chin Gen Pract Nurs 2014;12:351–2.

[12] Ding ZY Perioperative nursing of breast cancer patients who undergone DIEP. Nurs J Chin People's Liber Army 2006;12:67–8.

[13] Cao RJ Investigation of sexual status of patients who undergone mastectomy. Today Nurse 2011;114–6.

[14] Ren HL Jia XJ Wang Q Correlation between postoperative self-image and coping style of patients with breast cancer. Chin J Mod Nurs 2014;20:1274–7.

[15] Ghio L Gotelli S Cervetti A Duration of untreated depression influences clinical outcomes and disability. J Affect Disord 2015;175:224–8. 25658495 10.1016/j.jad.2015.01.014

[16] Li R Effective analysis of different surgical treatments in early-stage breast cancer. Chin Youjiang Med J 2013;41:4–6.

[17] Zhang HX Analysis of influential factors on quality of life of patients with breast cancer. Nurs J Chin People's Liber Army 2007;24:45–6.

[18] Huang LP Xiong BQ Application of high quality nursing on prostheses wearing in postoperative patients with breast cancer. J Yangtze Univ (Nat Sci Ed) 2013;10:42–3.

[19] Gallagher P Buckmaster A O’Carroll S Experiences in the provision, fitting and supply of external breast prostheses: findings from a national survey. Eur J Cancer Care 2009;6:556–68. 10.1111/j.1365-2354.2007.00898.x 19489989

[20] Thijs-Boer FM Thijs JT Van HB Conventional or adhesive external breast prosthesis? A prospective study of the patients’ preference after mastectomy. Cancer Nurs 2001;3:227–30. 11409067

[21] Kubon TM McClennen J Fitch MI A mixed-methods cohort study to determine perceived patient benefit in providing custom breast prostheses. Curr Oncol 2012;2:43–52. 10.3747/co.19.851 PMC3320231 22514496

[22] Li WH Zhang DM Wan CH Scales of quality of life of breast cancer patients. In: Zhang ZJ (Eds.), Handbook of behavioral medicine scale[M]. Editor, Zhang ZJ. Chinese medical electronic audio and video publishing press, 2005, 142-143.

[23] Hopwood P Fletcher I Lee A A body image scale for use with cancer patients. Eur J Cancer 2001;2:189–97. 10.1016/s0959-8049(00)00353-1 11166145

[24] Fang S Chang H Shu B Objectified body consciousness, body image discomfort, and depressive symptoms among breast cancer survivors in Taiwan. Psychol Women Q 2014;4:563–74.

[25] Ciesla S Polom K The effect of immediate breast reconstruction with Becker-25 prosthesis on the preservation of proper body posture in patients after mastectomy. Eur J Surg Oncol 2010;7:625–31. 10.1016/j.ejso.2010.05.005 20510569

[26] Rostkowska E Bak M Samborski W Body posture in women after mastectomy and its changes as a result of rehabilitation. Adv Med Sci 2006;51:287–97. 17357328

[27] Gallagher P Buckmaster A O’Carroll S External breast prostheses in post-mastectomy care: women's qualitative accounts. Eur J Cancer Care 2010;1:61–71. 10.1111/j.1365-2354.2008.00942.x 19708927

[28] Arroyo JM López ML Psychological problems derived from mastectomy: a qualitative study. Int J Surg Oncol 2011;2011:1–8. 10.1155/2011/132461 PMC3265278 22312492

[29] Duarte PT Andrade NA Enfrentando a mastectomia: analise dos relatos de mulheres mastectomizadas sobre questoes ligadas a sexualidade. Estud Psicol 2003;8:155–63.

[30] Breast Prostheses, Bras and Clothes After Surgery, Braille, DAISY. Available at: www.breastcancercare.org.uk/publications, 2014.

[31] Borghesan DHP Gravena AAF Lopes TCR Variables that affect the satisfaction of Brazilian women with external breast prostheses after mastectomy. Asian Pac J Cancer Prev 2014;22:9631–4. 10.7314/apjcp.2014.15.22.9631 25520080

[32] Münstedt K Schüttler B Milch W Epicutaneous breast forms. A new system promises to improve body image after mastectomy. Support Care Cancer 1998;3:295–9. 10.1007/s005200050170 9629886

[33] Koutz CA Quarnstrom MA Breast reconstruction after mastectomy. Clin Rev 2000;10:95–107.

[34] Jetha ZA Gul RB Lalani S Women experiences of using external breast prosthesis after mastectomy. Asia Pac J Oncol Nurs 2017;3:250–8. 10.4103/apjon.apjon_25_17 PMC5473097 28695172

[35] Glaus SW Carlson GW Long-term role of external breast prostheses after total mastectomy. Breast J 2009;4:385–93. 10.1111/j.1524-4741.2009.00742.x 19601944

[36] Hackethal A Münstedt K Shoulder strain caused by mammary prostheses ean experimental comparison of different forms of epicutaneous prostheses. Breast Care 2008;2:107–8. 10.1159/000210541 PMC2931070 20847888

[37] Kalpakcioglu B Fink M Tsiokas A Evaluation of the adhesive prosthesis “silima direct”by mastectomyzed patients in France. Marmara Med J 2008;2:133–45.

[38] Livingston PM White VM Roberts SB Women's satisfaction with their breast prosthesis: what determines quality prosthesis? Eval Rev 2005;29:65–83. 15604120 10.1177/0193841X04269640

[39] Roberts S Livingston P White V External breast prosthesis use: experiences and views of women with breast cancer, breast care nurses, and prosthesis fitters. Cancer Nurs 2003;26:179–86. 12832950 10.1097/00002820-200306000-00002

[40] Mahon S Casey M Patient education for women being fitted for breast prosthesis. Clin J Oncol Nurs 2003;2:194–9. 10.1188/03.CJON.194-199 12696216

[41] Liang YN Xu B Factors influencing utilization and satisfaction with external breast prosthesis in patients with mastectomy: a systematic review. Int J Nurs Sci 2015;2:218–24.

[42] Sun LQ Investigation of breast prosthesis among patients with mastectomy. J QILU Nurs 2010;21:48–9.

[43] Wang Y Yue HY Tan M The mental characteristics and nursing care for patients who use external breast prosthesis after mastectomy. J Mod Oncol 2008;7:1256–7.

[44] Healey IR External breast prostheses: misinformation and false beliefs. MedGenMed 2003;5:36. 14600672

[45] Livingston P Roberts S White V Do women have equitable access to quality breast prosthesis services? Aust N Z J Public Health 2000;4:452–3. 10.1111/j.1467-842x.2000.tb01612.x 11011478

[46] Sun X Wang S Xia Y Predictive-trend-aware composition of web services with time-varying quality-of-service. IEEE Access 2020;8:1910–21.

